# Prevalence of visceral leishmaniasis among people with HIV: a systematic review and meta-analysis

**DOI:** 10.1007/s10096-022-04530-4

**Published:** 2022-11-24

**Authors:** Maria Kantzanou, Maria A. Karalexi, Kalliopi Theodoridou, Evangelos Kostares, Georgia Kostare, Thalia Loka, Georgia Vrioni, Athanassios Tsakris

**Affiliations:** 1 Department of Microbiology, Medical School, National and Kapodistrian University of Athens, 115 27 Athens, Greece; 2 Department of Microbiology, Andreas Syggros Hospital for Skin and Venereal Diseases, National and Kapodistrian University of Athens, 161 21 Athens, Greece

**Keywords:** *Leishmania*, HIV, Coinfection, Prevalence, Systematic review, Meta-analysis

## Abstract

Leishmaniasis is a parasitic infection expressing different clinical phenotypes. Visceral leishmaniasis (VL) is considered an opportunistic infection among people with human immunodeficiency virus (HIV). The objective of this review was to identify published data on the prevalence of *Leishmania* spp. infection among PWH and to define particular determinants that affect critically the epidemiological characteristics of VL-HIV coinfection and, potentially, its burden on public health. Two independent reviewers conducted a systematic literature search until June 30, 2022. Meta-analyses were conducted using random-effects models to calculate the summary prevalence and respective 95% confidence intervals (CI) of leishmaniasis among PWH. Meta-regression analysis was performed to investigate the impact of putative effect modifiers, such as the mean CD4 cell count, on the major findings. Thirty-four studies were eligible, yielding a summary prevalence of 6% (95%CI, 4–11%) for leishmaniasis (*n* = 1583) among PWH (*n* = 85,076). Higher prevalence rates were noted in Asia (17%, 95%CI, 9–30%) and America (9%, 95%CI, 5–17%) than in Europe (4%, 95%CI, 2–8%). Prevalence rates were significantly mediated by the age, sex, and CD4 cell count of participants. Heterogeneity remained significant in all meta-analyses (*p* < 0.0001). In the majority of included studies, people were coinfected with HIV and *Leishmania* species associated with VL, as opposed to those associated with cutaneous leishmaniasis. No sign of publication bias was shown (*p* = 0.06). Our summary of published studies on leishmaniasis among PWH is important to provide prevalence estimates and define potential underlying factors that could guide researchers to generate and further explore specific etiologic hypotheses.

**Supplementary Information:**

The online version contains supplementary material available at 10.1007/s10096-022-04530-4.

## Introduction

Leishmaniasis is a vector-borne parasitic infection with three primary clinical forms: visceral leishmaniasis (VL), cutaneous leishmaniasis, and mucocutaneous leishmaniasis [1]. More than 20 distinct species of the genus *Leishmania* are responsible for the clinical manifestations of the disease. The global incidence of leishmaniasis is estimated at 700,000 to 1 million new cases per year [2, 3]. Though the clinical manifestations of *Leishmania* infection are traditionally classified into three syndromes, immunocompromised people are more likely to have disseminated disease [1]. *Leishmania* species that typically cause only cutaneous disease could cause disseminated disease in people with compromised cellular immunity such as people with human immunodeficiency virus (HIV (PWH)).

Over the last decade, advances in healthcare delivery have led to substantial decreasing trends in VL although it remains a fatal disease in endemic areas, such as in some African regions [2, 4]. VL is considered an opportunistic infection for PWH in countries/regions, where leishmaniasis is endemic because coinfection rates are much higher in *Leishmania*-endemic countries/regions [5]. As of 2021, *Leishmania*-HIV coinfections have been reported from 45 countries [6, 7]. High *Leishmania*-HIV coinfection rates have been noted in Brazil, Ethiopia, and the state of Bihar in India [8–11]. *Leishmania*-HIV coinfected people are more likely to develop the disseminated clinical disease, as well as high relapse and mortality rates [6]. Due to the high prevalence of *Leishmania*-HIV coinfection in these countries and the lack of a single reliable serologic test for leishmaniasis diagnosis, screening for *Leishmania* coinfection with a combination of laboratory tests would be useful for PWH living in or originating from endemic regions [12]. From a therapeutic perspective, the critical role of highly active antiretroviral therapy (HAART) in the outcome of VL-HIV coinfection has been clearly reported [13]. In particular, once *Leishmania*-HIV coinfection is diagnosed, early initiation of HAART is recommended as a protective modality against VL relapses. Antiretroviral treatment reduces the development of the disease, delays relapses, and increases the survival of the coinfected patients [14]. Several reviews have synthesized the existing evidence around the prevalence of *Leishmania*-HIV coinfection, as well as the demographic and diagnostic characteristics of the disease [15–17]. For instance, Lindoso et al. reported that VL occurs in 12 countries of Latin America, with 96% of cases reported in Brazil [15]. Moreover, Boelaert et al. showed that specific immunochromatographic test, i.e., rK39 ICT, shows high sensitivity and specificity for the diagnosis of visceral leishmaniasis in patients with febrile splenomegaly and no previous history of the disease, but the sensitivity is notably lower in east Africa than in the Indian subcontinent [16].

Overall, the results of the present study is expected to be useful to public health authorities when implementing better control strategies and guiding future developments in the field. We reviewed published evidence on the prevalence rates of *Leishmania* spp. among PWH and to quantitatively synthesize the results of published studies.

## Methods

### Search strategy and study selection

A literature search of Medline database was conducted from inception up to June 30th, 2022, following the Preferred Reporting Items for Systematic Reviews and Meta-Analyses (PRISMA) guidelines (Supplementary Table 1) [18]. Two reviewers performed the literature search independently using the algorithm (leishmania OR leishmaniasis) AND (immunocompromised OR immunodeficiency OR HIV OR AIDS OR (immune AND deficiency)) AND (incidence OR prevalence OR rate). Both reviewers evaluated the reference lists of all identified eligible studies using the “snowball” procedure, which entails searching the reference lists of each manuscript for additional eligible articles potentially missed from the initial literature search. We included articles that examined specifically the prevalence rates of leishmaniasis among PWH. No language or other restrictions were applied. Case reports and experimental or animal studies were excluded. Following the literature search, duplicate citations were removed, and the remaining articles were independently screened by two investigators to identify studies that met the pre-determined inclusion criteria. The study selection was conducted in two stages. First, we reviewed article titles and abstracts and removed those that did not meet our inclusion/exclusion criteria. Second, we retrieved and reviewed the full text of the remaining articles. For the remaining studies, the full papers were retrieved for further screening. In case of disagreement in the selection of studies or snowball procedure, the final decision was reached by team consensus. In articles with overlapping populations, the most recent or most complete publication was considered eligible.

### Data extraction

For each eligible publication, the following study variables were extracted: publication year, country/region where the study was performed, study design, study period, follow-up period, sample size, age at diagnosis, mean patient’s CD4 count, and the proportion of males. In addition, information about the number of patients with *Leishmania*-HIV coinfection, the type of *Leishmania* infection, type of *Leishmania* diagnostic method, the treatment administered, and the outcome of such infection were also extracted. Two reviewers performed the data extraction, and any disagreements were resolved by consensus.

### Statistical analysis

A descriptive presentation of the eligible studies was initially performed (Table 1). Thereafter, the prevalence of *Leishmania* infection among PWH and the respective 95% confidence intervals (CI) were extracted or calculated from the available data using the Wilson’s method [19]. Meta-analyses were undertaken using random-effects models [20] to estimate the prevalence of *Leishmania*-HIV coinfection overall and by study region (Europe, America, and Asia). We performed sub-analyses of prevalence by region where numbers allowed. Between-study heterogeneity was assessed using the Cochran *Q* and *I*^2^ statistics^20^. The *Z*-test was applied for the overall effect, and statistical significance was set at *p* < 0.10. In addition, we performed sensitivity meta-analyses excluding a study per time to identify whether heterogeneity was affected by the inclusion of a specific study. The impact of potential effect modifiers, such as publication year, mean age, percent of males, and mean CD4 cell count on the main results, was explored in meta-regression analysis. Publication bias was calculated using the Egger’s test (significance level was set at *p* < 0.05) [21]. Analyses were performed using the Stata software.

**Table 1:** Descriptive characteristics of eligible studies

Study	Study design	Region	Study period	Follow-up (mean, years)	*N* patients	% males	Mean age	CD4 cell count (mean) cells/mm^−3^	Leishmaniasis (*N*)	Type of *Leishmania*	Ascertainment of outcome	Treatment
Bissuel (1994) [53]	Cohort	France	1989–1991	2.5	270	100	NR	94	4	NR	BM culture	NR
Dereure (1995) [51]	Cohort	France	1989–1993	NR	139	NR	NR	NR	10	Visceral	BM aspirate	NR
Del Giudice, (2002) [42]	Cohort	France	1992–1999	3.2	55.626	75	39.1	133	165	Visceral	BM, blood, visceral samples	Antiretroviral, PI
Gradoni (1996) [50]	Cohort	Italy	1989–1994	NR	318	NR	NR	NR	10	NR	BM, blood, skin biopsy	Conventional pentavalent antimony or liposomal amphotericin B pentamidine allopurinol itraconazole aminosidine
Russo (2003) [41]	Cohort	Italy	1990–1998	NR	620	82	33.5	62	27	Visceral (25 patients), Cutaneous (2)	BM biopsy in 26L, lymph node biopsy in 1L	Pentavalent antimonial compounds, amphotericin B, lipidic formulation
Colomba (2009) [37]	Cross-sectional	Italy	2008	NR	145	68	43	432	24	Visceral	Blood samples	Liposomal amphotericin B
Miralles (1995) [52]	Cohort	Spain	1991–1992	1.3	580	84	30	71	7	Visceral	BM biopsy	NR
Amela (1996) [54]	Cohort	Spain	1982–1993	NR	6652	NR	NR	NR	166	Visceral	NR	NR
Lozano (1996) [49]	Cohort	Spain	1992–1993	NR	3603	87	30	46	23	Visceral	BM aspirate, liver biopsy	NR
Benito (1997) [48]	Cohort	Spain	1991–1995	NR	123	84	36	NR	4	Visceral	BM biopsy	NR
Pineda (1998) [47]	Cross-sectional	Spain	1993–1997	NR	291	82	32	176	32	Visceral	BM aspirate	NR
Dereure (1998) [46]	Cohort	Mediterranean^a^	1993–1996	NR	65	NR	NR	NR	12	Visceral	Blood, BM	NR
Bernabeu-Wittel (1999) [45]	Cohort	Spain	1996–1997	0.5	32	91	33	40	7	Visceral	BM	NR
Pintado, 2001 [44]	Cross-sectional	Spain	1986–1997	NR	7.438	NR	NR	NR	81	Visceral	Blood, tissue samples	Meglumine antimoniate combine with allopurinol or interferon γ, amphotericin B deoxycholate or liposomal amphotericin B, pentamidine isethionate, allopurinol ketoconazole itraconazole
De La Rosa, 2002 [43]	Cohort	Spain	1989–2000	3.8	479	76	31	294	21	Visceral	BM aspirate	HAART
Garcia-Garcia, 2006 [39]	Cross-sectional	Spain	2004	NR	92	75	40,9	419	28	NR	Blood, urine samples, skin test	NR
Abellán-Martínez, 2009 [35]	Cohort	Spain	1994–2000	NR	276	79	32	28	27	NR	NR	NR
Ena, 2014 [31]	Cross-sectional	Spain	2009–2012	1	179	80	NR	NR	7	Visceral	BM, tissue biopsy, urine antigen, skin biopsy	Liposomal amphotericin B in subclinical and symptomatically
Soares, 2008 [38]	Cohort	Brazil	2001–2004	NR	828	71	35.4	NR	68	Visceral	NR	NR
Carranza-Tamayo, 2009 [36]	Cross-sectional	Brazil	2005–2006	NR	163	69	37	314	26	Visceral	BM aspirate, Blood samples	NR
Nascimento (2011) [34]	Cross-sectional	Brazil	1990–2009	NR	3157	88	37.3	86	17	Visceral	BM aspirate, Blood samples	Amphotericin B
Orsini (2012) [33]	Cross-sectional	Brazil	1999–2002	1.2	381	64	34.2	384	77	Visceral	Blood samples	NR
Guedes (2018) [28]	Cross-sectional	Brazil	2014–2015	NR	207	68	39.7	219	35	Visceral	BM(18), urine, blood samples	NR
Walter Lins Barbosa Junior (2020) [24]	Cross-sectional	Brazil	2012–2017	NR	309	67	36	269	110	Visceral	Blood samples, BM	NR
Cunha (2020) [22]	Cohort	Brazil	2015–2016	NR	240	72	45.5	564.4	36	Visceral	Blood samples	NR
Guedes (2021) [12]	Cross-sectional	Brazil	NR	NR	483	61	NR	NR	44	Visceral	Blood, urine samples	NR
Salvador (2013) [32]	Cohort	America, Africa^b^	2010–2011	NR	190	68	37	459	7	NR	BM aspirate, Blood samples	NR
Echchakery (2018) [25]	Cohort	Morocco	2016	0.5	200	42	NR	NR	10	Visceral	Blood samples	NR
Shafiei (2014) [30]	Cohort	Iran	2011–2012	NR	49	65	43.7	236.22	9	Visceral	BM aspirate	CMX, AZIT
Rezaei (2018) [26]	Cross-sectional	Iran	2017	NR	251	64	NR	404	19	Visceral	Blood samples	NR
Sharma (2004) [40]	Cross-sectional	India	2000–2003	NR	135	83	34	121	2	Visceral	NR	Antiretroviral, NNRTI
Pandey (2018) [27]	Cross-sectional	Thailand	2015–2016	0.5	305	49	43.2	NR	176	Visceral	Blood, saliva samples	NR
Manomat (2017) [29]	Cross-sectional	Thailand	2015–2016	0.5	724	52	43.6	NR	182	Visceral	Blood sample	Amphotericin B
Charoensakulchai (2020) [7]	Cross-sectional	Thailand	2015–2016	0.5	526	53	43.9	NR	110	NR	Blood, saliva samples	NR

^*^NR, not reported; *BM*, bone marrow; *NNRTI*, non-nucleoside reverse transcriptase inhibitor; *PI*, protease inhibitor; *HAART*, highly active antiretroviral therapy; *CMX*, cotrimoxazole; *AZIT*, azithromycin

^a^the country of origin and the nationality of the patients is not defined

^b^a total of 190 patients were included: 141 (74.2%) from Latin America, 41 (21.6%) from sub-Saharan Africa, and 8 (4.2%) from northern Africa

## Results

### Characteristics of the studies

**Fig. 1:**
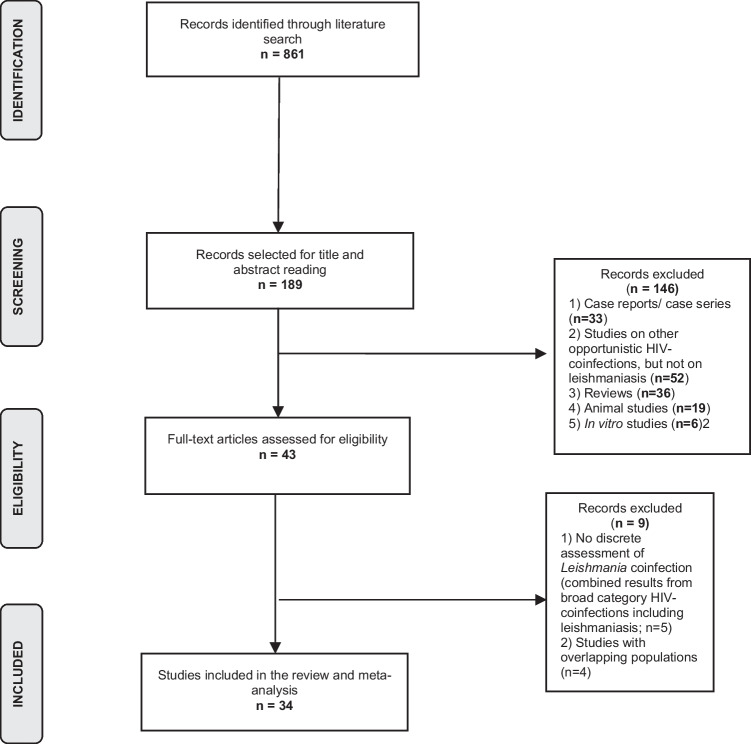
Flow chart presenting the selection of the eligible studies

Figure 1 shows the results of the literature search and selection process. Following the exclusion of publications with overlapping populations, a total of 34 eligible studies were finally included in this analysis [12, 22–54].

The descriptive characteristics of the included studies are presented in Table 1. Nineteen of the studies were of cohort design, and the remaining fifteen were found to be of cross-sectional design. The mean follow-up period ranged between 0.5 and 3.8 years. Most studies were conducted in Europe (France, Spain, Italy; *n* = 18), followed by America (Brazil, USA; *n* = 9), Asia (Iran, Thailand; *n* = 6), and Africa (Morocco; *n* = 1).

### Prevalence of leishmaniasis among PWH

**Fig. 2:**
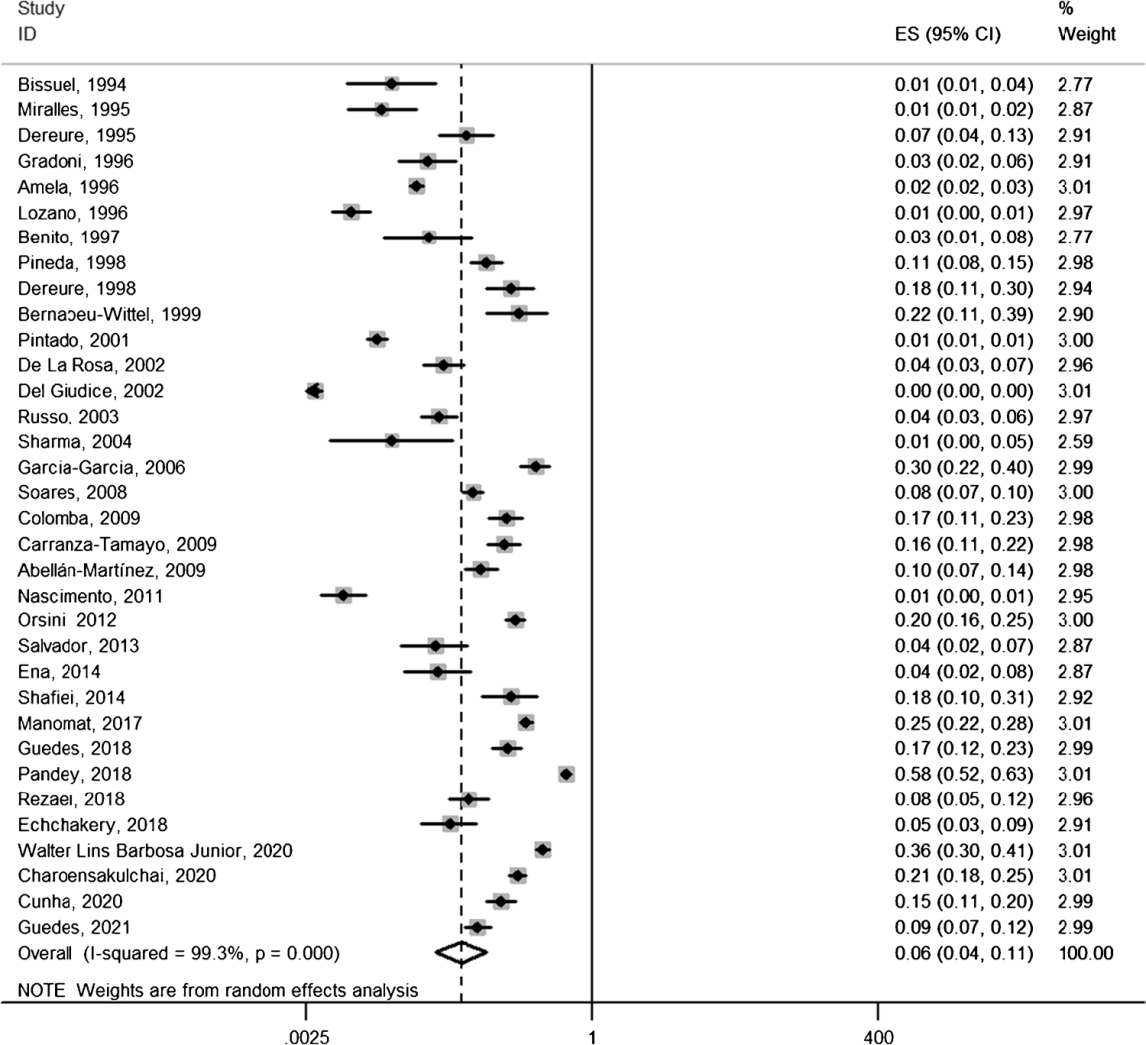
Forest plot showing the prevalence of *Leishmania*-HIV coinfection. Prevalence ratios of individual studies are indicated by the data markers; shaded boxes around data markers reflect the statistical weight of the study; 95% confidence intervals (CI) are indicated by the error bars; summary-effect estimates with their 95% CI are depicted as a diamond

**Fig. 3:**
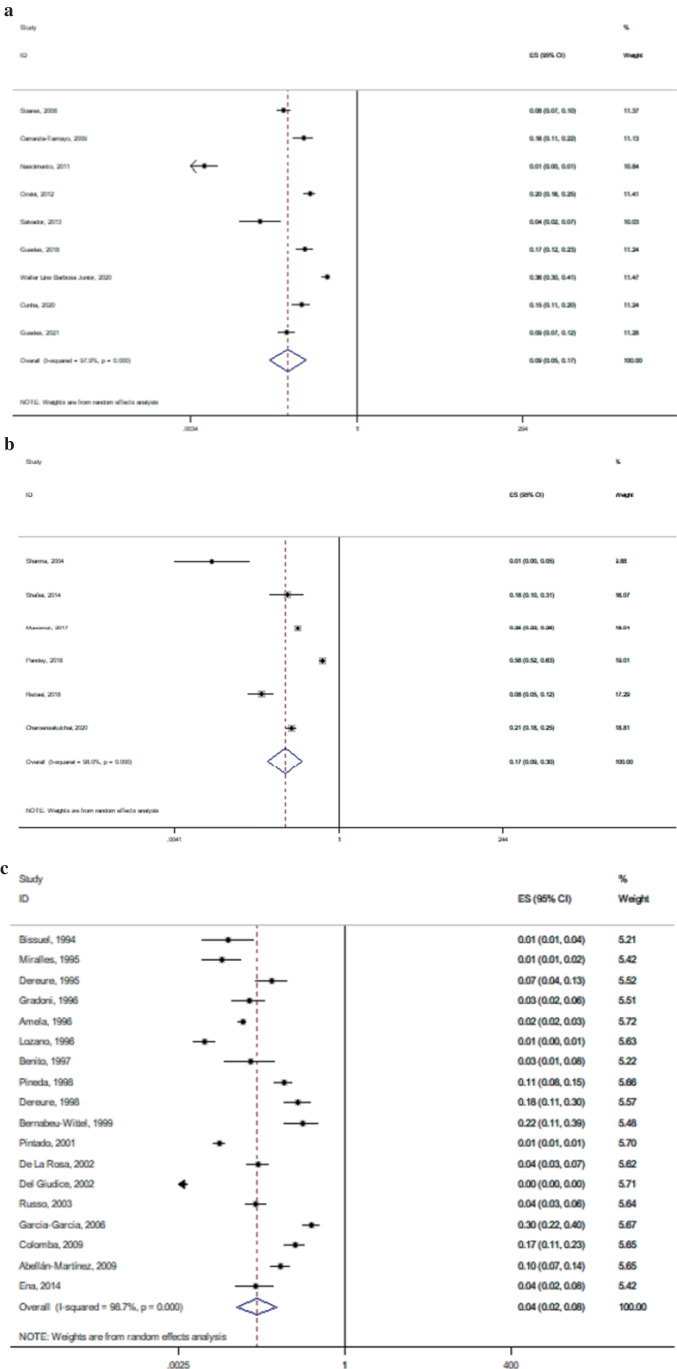
Forest plot showing the prevalence of *Leishmania*-HIV coinfection by study origin: **a** America, **b** Asia, and **c** Europe. Prevalence ratios of individual studies are indicated by the data markers; shaded boxes around data markers reflect the statistical weight of the study; 95% confidence intervals (CI) are indicated by the error bars; summary-effect estimates with their 95% CI are depicted as a diamond

Thirty-four eligible studies yielded a total sample of 85,076 PWH, of which leishmaniasis was diagnosed in 1583 people. The average percent of males was 72.4%, while the mean age of participants ranged from 30 to 41 years (median 36.9 years). Diagnosis of leishmaniasis was established through bone marrow biopsy and/or blood tests in the majority of studies (Table 1). The prevalence of leishmaniasis was found to be particularly variable across regions globally, although a summary prevalence of leishmaniasis among PWH was estimated as high as 6% (95% CI, 4–11%; *n* = 34 studies) with significant between-study heterogeneity (*I*^2^, 99.3%, *p* < 0.0001) (Fig. 2). The prevalence was 9% among studies conducted in America (95% CI, 5–17%; *n* = 9 studies) (Fig. 3a) and even higher among those conducted in Asia (17%, 95% CI, 9–30%; *n* = 6 studies) (Fig. 3b) compared to the prevalence calculated for European studies (4%, 95% CI, 2–8%; *n* = 18 studies) (Fig. 3c). Heterogeneity remained significant in the subgroup meta-analyses by study origin.

In the majority of included studies, people were diagnosed with HIV and *Leishmania* species associated with VL, as opposed to *Leishmania* species associated with cutaneous leishmaniasis, with the exception of one study where 25 out of 27 *Leishmania*-HIV infected participants had VL, and the remaining two had cutaneous leishmaniasis [41].

In the meta-regression analyses, age (*p* = 0.05) and CD4 cell count (*p* = 0.02) were statistically significant positive modifiers of the observed associations, whereas sex (percent of males) had a significant inverse effect modification (*p* = 0.002). Publication year had a borderline mediating effect on the results of the main meta-analyses (*p* = 0.06) (Table 2). Sensitivity meta-analyses excluding a study per time showed similar results with those of the main analyses, without reducing the between-study heterogeneity. Egger’s test showed no sign of publication bias (*p* = 0.06) (Supplementary Fig. 1). Information regarding treatment of *Leishmania*-HIV individuals was reported in 11 studies only. Liposomal amphotericin B, conventional pentavalent antimonial, pentamidine, allopurinol, itraconazole, and aminosidine were reported as medications; amphotericin B was the most frequently used medication.

**Table 2:** Results of meta-regression analysis examining the potential modifying role of publication year, age, and percentage of males in the individual studies

	*n*^§^	Exponentiated coefficient (95%CI)	*p*
Publication year (10-year increments)	33	1.69 (0.99–2.87)	0.06
Percentage of males	29	0.95 (0.91–0.98)	0.002
Mean age	24	1.07 (1.00–1.14)	0.05
Mean CD4 cell count	21	1.01 (1.00–1.02)	0.02

^§^number of study arms

## Discussion

The present systematic review and meta-analysis of 34 published studies including 1583 individuals with *Leishmania*-HIV coinfection provides evidence for 6% prevalence of leishmaniasis among PWH living in or from populations of *Leishmania*-endemic regions. It should not be overlooked, however, that the abovementioned prevalence practically reflects the overall frequency in VL-PWH in the specific countries identified and included in the present study. As it was expected, we observed higher prevalence in studies from Asia and the Americas compared to studies from Europe; data on leishmaniasis among PWH were unavailable for other regions where *Leishmania* is endemic including Africa and the Middle East. In addition, the present results are consistent with the already well-described caveat that the prevalence of species causing VL is higher in regions (i.e., the “Old World”) where such species are endemic and regions with migration from areas where such species are endemic. Similarly, we found the prevalence of cutaneous and mucocutaneous leishmaniasis to be the highest in regions where the species causing these disease manifestations are endemic (e.g., the “New World”).

Prevalence rates varied significantly by age, sex, and CD4 cell count of participants. Publication year also had a borderline significant mediating effect, which could be potentially explained by treatment advances, i.e., the increased availability of ART as time progressed. Overall, the results were based on highly heterogeneous studies mainly conducted in Europe. Indeed, the variation of findings by the study setting in variable time periods or different counties points to a very complex interplay between a wide range of environmental and genetic factors.

Leishmaniasis is considered an opportunistic infection among PWH, especially among those living in VL-endemic sub-tropical and tropical regions around the world, including the Mediterranean [55]. Globally, the occurrence of *Leishmania*-HIV coinfection has been enhanced by the spread of HIV into rural areas and the concurrent spread of leishmaniasis to suburban/urban areas [56, 57]. Some experts expect the number of cases of *Leishmania*-HIV coinfection will increase as the distributions of the two infections further overlap [58]. In several Southwestern European countries, such as France, Italy, Spain, and Portugal, increasing incidence rates of *Leishmania*-HIV coinfection have been noted, with most cases concerning VL [57].

Studies on the immunological background of *Leishmania*-HIV coinfection report that both microorganisms infect and multiply within myeloid or lymphoid cells, enhancing thus the reciprocal modulation of *Leishmania* and HIV pathogenesis [57]. Additionally, given that recovery from leishmaniasis is associated with long-term persistence of parasites at the primary sites of infection and their draining lymph nodes, it is likely that HIV-mediated immunosuppression due to CD4( +) T cell depletion could lead to reactivation leishmaniasis, particularly in immunocompromised patients [59–61]. Indeed, the present results suggest a potential prevalence effect modification due to the PWH population control of HIV infection, which CD4 count is a surrogate marker for. Of note, the mean CD4 count for nearly all cohorts was less than 200, a finding clearly carrying increased risk of leishmaniasis clinical manifestations.

The lethality of VL increases by 4.6% to 16.6% when associated with HIV infection [62]. The presence of HIV can lead to severe forms of VL, which are difficult to control and manage. Furthermore, PWH are more likely to have severe and/or prolonged VL with a more difficulty treatment course, which further may increase the HIV replication and the clinical evolution of AIDS [62]. Therefore, this coinfection warrants concern and must be recognized and treated in a timely manner. Recent reports from the World Health Organization recommend that patients with *Leishmania*-HIV coinfection should be treated with liposomal amphotericin B, whereas early administration of HAART acts as a protective modality against VL relapse [6, 55].

The sound methodological approach has been a strength of the current study given that meta-analyses, sub-analyses by study origin, sensitivity meta-analyses, and meta-regression analyses were run. However, the present results should be cautiously interpreted in view of limitations inherent to the study design and data availability of eligible studies including variable criteria for selection of the comparison groups, statistical analysis methods, and variable follow-up periods.

The highly heterogeneous results across the included studies could either indicate that different factors may be implemented in the prevalence of *Leishmania*-HIV coinfection in each country or could point to the heterogeneity of the methodological approaches, e.g., in statistical analysis, implemented by each study. Moreover, information on other variables of interest, such as ART at time of leishmaniasis diagnosis, mortality of people with *Leishmania*-HIV coinfection, and use of secondary chemoprophylaxis post-leishmaniasis treatment, were available by only 1–2 studies, thus not allowing generalization of the results.

Although the articles included in the present study are biased towards specific regions (Europe and Brazil) and cases visceral leishmaniasis only, it becomes evident that the field of VL in HIV patients remains of particular scientific interest. Further studies, both retrospective and prospective, should be specially designed and conducted in order the role of critical individual determinants of the disease to be defined and the between-country comparisons to be feasible.

## Conclusions

In view of the still rising rates of the HIV/AIDS pandemic in some parts of the world, we noted a high prevalence of *Leishmania* infection among PWH. Heterogeneity issues and diverse study settings should be taken into account when interpreting the results of the present study. However, and beyond the above factors, this attempt to summarize current findings is of significant importance in providing effect estimates, as well as to point towards potential underlying factors. Further research based on well-designed studies is needed to generate and explore specific etiological hypotheses, as well as to compare prevalence and determinants between countries reliably and effectively.

## Supplementary Information

Below is the link to the electronic supplementary material.

Supplementary file1 (DOCX 51 KB)

## Data Availability

The datasets generated during and/or analyzed during the current study are not publicly available due to potential compromise of individual privacy but are available from the corresponding author on reasonable request.

## Code availability

Not applicable for this submission.
